# A pilot study of contrast-enhanced electrical impedance tomography for real-time imaging of cerebral perfusion

**DOI:** 10.3389/fnins.2022.1027948

**Published:** 2022-11-24

**Authors:** Yuyan Zhang, Jian’an Ye, Yang Jiao, Weirui Zhang, Tao Zhang, Xiang Tian, Xuetao Shi, Feng Fu, Liang Wang, Canhua Xu

**Affiliations:** ^1^College of Life Sciences, Northwest University, Xi’an, China; ^2^Department of Biomedical Engineering, Fourth Military Medical University, Xi’an, China; ^3^Shaanxi Provincial Key Laboratory of Bioelectromagnetic Detection and Intelligent Perception, Xi’an, China; ^4^Department of Neurosurgery, Tangdu Hospital of Fourth Military Medical University, Xi’an, China

**Keywords:** electrical impedance tomography, cerebral perfusion, angiography, imaging, rabbits

## Abstract

**Background:**

Real-time detection of cerebral blood perfusion can prevent adverse reactions, such as cerebral infarction and neuronal apoptosis. Our previous clinical trial have shown that the infusion of therapeutic fluid can significantly change the impedance distribution in the brain. However, whether this alteration implicates the cerebral blood perfusion remains unclear. To explore the feasibility of monitoring cerebral blood perfusion, the present pilot study established a novel cerebral contrast-enhanced electrical impedance tomography (C-EIT) technique.

**Materials and methods:**

Rabbits were randomly divided into two groups: the internal carotid artery non-occlusion (ICAN) and internal carotid artery occlusion (ICAO) groups. Both of groups were injected with glucose, an electrical impedance-enhanced contrast agent, through the right internal carotid artery under EIT monitoring. The C-EIT reconstruction images of the rabbits brain were analyzed according to the collected raw data. The paired and independent *t*-tests were used to analyze the remodeled impedance values of the left and right cerebral hemispheres within and between studied groups, respectively. Moreover, pathological examinations of brain were performed immediately after C-EIT monitoring.

**Results:**

According to the reconstructed images, the impedance value of the left cerebral hemisphere in the ICAN group did not change significantly, whereas the impedance value of the right cerebral hemisphere gradually increased, reaching a peak at approximately 10 s followed by gradually decreased. In the ICAO group, the impedance values of both cerebral hemispheres increased gradually and then began to decrease after reaching the peak value. According to the paired *t*-test, there was a significant difference (*P <* 0.001) in the remodeling impedance values between the left and right hemispheres in the ICAN group, and there was also a significant difference (*P* < 0.001) in the ICAO group. According to the independent *t*-test, there was a significant difference (*P* < 0.001) of the left hemispheres between the ICAN and ICAO groups.

**Conclusion:**

The cerebral C-EIT proposed in this pilot study can reflect cerebral blood perfusion. This method has potential in various applications in the brain in the future, including disease progression monitoring, collateral circulation judgment, tumor-specific detection, and brain function research.

## Introduction

Cerebral perfusion plays a particularly important role as it reflects the blood supply provided by cerebral blood vessels (Leng et al., 2016). Good cerebral perfusion is conducive to maintaining the normal function of the brain, and stenosis or occlusion of blood vessels supplying the brain will lead to insufficient cerebral perfusion (Clayton et al., 2008; Ginsberg, 2018). When cerebral ischemia is severe and compensatory blood flow is insufficient, a series of reactions that are especially injurious to neurons are triggered (Pivonkova et al., 2010; Ofengeim et al., 2011). Furthermore, studies have shown that neurons die in ischemic conditions in approximately half the time as non-neural cells, indicating ischemia and subsequent hypoxia are extremely damaging to neural tissue (Song and Yu, 2014; Roh and Kim, 2016). Therefore, it is particularly important to monitor cerebral blood perfusion in real time during surgery and other procedures to prevent adverse events, such as neuronal apoptosis or brain tissue infarction.

Cerebral perfusion imaging evaluates regional microvascular hemodynamics in the living brain, allowing for *in-vivo* measurement of a variety of hemodynamic parameters. Clinical perfusion imaging techniques typically rely on X-ray computed tomography (CT) or magnetic resonance imaging (MRI), which is called CT perfusion (CTP) and MR perfusion (MRP), respectively (Copen et al., 2016). CTP and MRP have been used as a routine examination and clinical standard of care for stroke (Vagal et al., 2019). In addition, PET scan, regarded as the “gold standard” for the blood perfusion imaging, can provide information of cerebral blood flow due to its capability of evaluating the uptake of nutrients in the blood flow (Heurling et al., 2017). However, these technologies all have their own shortcomings, such as bulky equipment, high price, scarce resources, difficulty in usage in local or rural hospitals. Therefore, there is an urgent need for a novel, lightweight, convenient, and low-cost cerebral blood perfusion imaging technology that can be used as a powerful assistant to the existing imaging technology.

Electrical impedance tomography (EIT) is a portable, economical, safe, and constant real-time imaging technology, which applies a safe excitation current to the human body through contact electrodes, and the resulting changes in impedance distribution allow reconstructed image analysis using algorithms (Holder, 2005; Adler and Boyle, 2017; Pan et al., 2020). Due to its advantages, such as small time interval for data collection, sensitivity to changes in electrical conductivity of different tissues, and that it requires no use of radiation, EIT has been widely applied in many fields, for instance, lung respiratory function imaging (Aguiar Santos et al., 2018; Tomicic and Cornejo, 2019), prostate cancer detection function imaging (Tan and Rossa, 2021), and abdominal organ function imaging (Shuai et al., 2009).

In recent years, EIT has become a research hotspot in the field of functional brain imaging. Sana et al. characterized and imaged certain impedance distribution changes during seizures in neocortex and hippocampus using the rat model. The results suggest that EIT can be used as an ancillary imaging method for conventional electroencephalogram to improve the localization of epileptogenic regions in patients with refractory epilepsy who undergo surgery to control seizures (Hannan et al., 2021). Using an intraoperative EIT system to monitor the brain impedance of patients, Li et al. (2018) found that brain impedance was negatively correlated with cerebral blood perfusion, and the slow rise in brain resistivity might reflect changes in brain tissue caused by ischemia. The results showed that changes in regional cerebral impedance could be detected by EIT, and these changes were larger with lower blood perfusion, suggesting that EIT is expected to reflect quantitative information about cerebral blood perfusion in certain regions (Li et al., 2018). However, the accuracy of traditional electrical impedance functional imaging is insufficient to reflect the impedance changes caused by heartbeats. Based on previous clinical experiments by our research group, the infusion of a therapeutic liquid can significantly change the impedance distribution of the brain. Therefore, we propose a new method of brain contrast-enhanced electrical impedance tomography (C-EIT), which fully utilizes the advantages of differential subtraction imaging to reflect cerebral blood perfusion.

In the study, we established a rabbit model of unilateral internal carotid artery occlusion (ICAO) and normal cerebral blood perfusion and further explored the one-dimensional impedance and reconstructed images of the left and right hemispheres after electrical impedance contrast perfusion to reflect cerebral blood perfusion. This study aimed to establish an animal model of internal carotid artery occlusion in rabbits to explore the feasibility of brain electrical impedance-enhanced contrast perfusion imaging to reflect cerebral blood perfusion.

## Materials and methods

### Animal preparation

All animals in this study were purchased from the Experimental Animal Center of Fourth Military Medical University. The experimental protocols and procedures were approved by the Animal Ethics Committee of the Fourth Military Medical University (Shaanxi, P. R. China) and complied with the “Guide for the Care and Use of Laboratory Animals” published by the National Institutes of Health (National Academy Press, Washington, DC, revised 1996). Twenty New Zealand white rabbits (male and female) weighing 2.5–3.0 kg were randomly divided into two groups: the internal carotid artery non-occlusion (ICAN) group (*n* = 10) and the ICAO group (*n* = 10). In the ICAO group, unilateral internal carotid artery clipping was controlled for 30 min to ensure adequate changes in cerebral perfusion.

**FIGURE 1:**
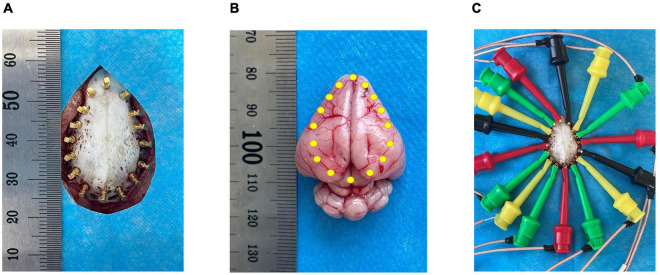
Schematic diagram of electrode positions. **(A)** Shows the 16 copper electrode positions on the skull; **(B)** Shows the relative position of the copper electrodes in the brain parenchyma; **(C)** Shows the electrode wire with a hook-shaped tool at the end connected to the copper electrode.

Before the experiment, an electric heating plate was used to maintain the animal’s body temperature to prevent death due to the loss of temperature caused by deep anesthesia. The animals were anesthetized with isoflurane gas (3% for induction and 2% for maintenance). The skin of the cranium was cut longitudinally with a sterile scalpel, the periosteum was removed, coronal and sagittal sutures were positioned, and a 4.0 × 2.5 cm area was exposed. Four sterile copper electrodes 0.12 cm in length were implanted 1.2 cm from the sagittal suture and 1.0 cm from the coronal suture. The remaining 12 copper electrodes were symmetrically and evenly spaced, and 16 copper electrodes formed an elliptical ring (Figure 1). None of the copper electrodes penetrated the intracranial dura mater in the skull. This operation ensured that the copper electrode was separated from the surrounding periosteum and skin to prevent the impedance value from being affected by skin contact.

The internal carotid artery was isolated after the electrodes were successfully implanted into the skull. The animal was supine and fixed to the dissection table; the skin and subcutaneous fascia of the middle of the neck were cut and the muscle was bluntly separated near the trachea. The bilateral common carotid arteries were exposed sequentially, and the dissection was continued upward to the position of the mastoid bone to expose the bifurcation of the internal carotid artery and the external carotid artery.

### Contrast-enhanced electrical impedance tomography monitoring

In this study, the in-house FMMU-EIT-5 system was used to collect the injection and metabolism signals of the internal carotid artery contrast agent. The frequency range of the system was 1–190 Hz, and the output current range was 10–1250 μ Ap-p (Cao et al., 2020). Studies have shown that a small interference of 0.35% volume (cross-sectional area of 1.99%) and 17% resistivity can be detected in human brain models (Shi et al., 2018). A special electrode wire wrapped with insulating material was connected to the copper electrode through the end hook to transmit the excitation current. A 500 μ Ap-p current stimulus was administered between two polar pairs of electrodes, and the voltage difference was measured on the remaining adjacent electrode pairs at a rate of one frame/sec (Xu et al., 2007).

**FIGURE 2:**
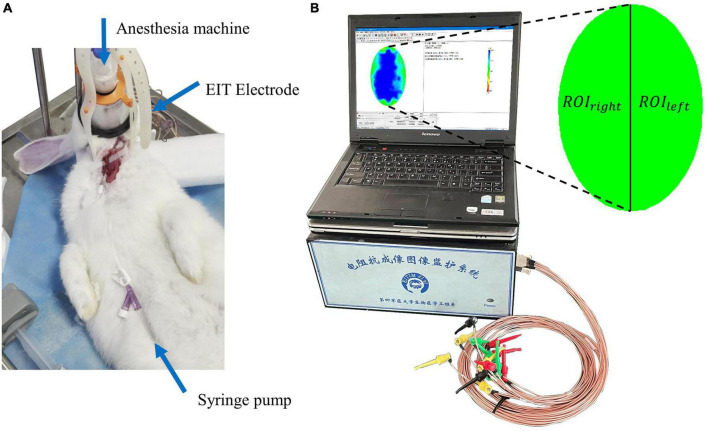
**(A)** The electrical impedance tomography (EIT) imaging experiment. **(B)** The FMMU-EIT5 monitoring system, where ROI_*left*_ refers to the region of interest in the left hemisphere, and ROI_*right*_ refers to the region of interest in the right hemisphere.

**FIGURE 3:**
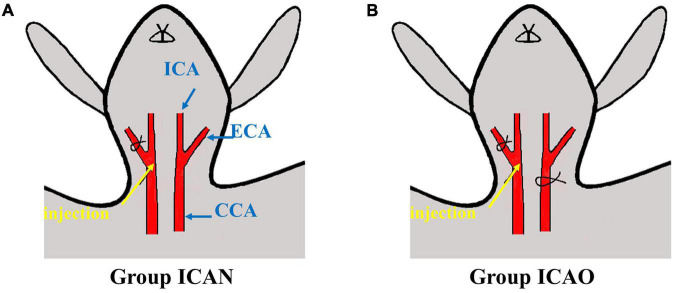
Schematic diagram of injection. **(A)** Indicates injection to the ICA with a normal CCA. **(B)** Represents injection to the ICA with occlusion of the CCA. (CCA, common carotid artery; ECA, external carotid artery; ICA, internal carotid artery).

After the cranial electrodes were successfully placed and connected to the FMMU-EIT-5 system, electrical impedance-enhanced contrast perfusion imaging was performed (Figure 2). The contrast medium uses 5% glucose injection, which is a common clinical isotonic agent, and its electrical conductivity (0.02 S/m) was measured by a four-electrode box and the Solartron 1260 + 1294 impedance analyzer (Schlumberger Company, Hampshire, UK) (Liu et al., 2019). This value is about 30 times different from the blood conductivity (0.67 S/m). For the ICAN group, after the right external carotid artery was ligated, a 26G needle was used to puncture near the branch of the right common carotid artery, and glucose was injected, while the left internal carotid artery was left intact (Figure 3A). The contrast agent glucose perfusion dose was 1.0 ml/kg, and the perfusion rate was 0.25 ml/s. For the ICAO group, the left common carotid artery was ligated using a surgical silk suture. The right external carotid artery was ligated near the branch of the right common carotid artery. A 26G puncture needle was then used to puncture the right common carotid artery at a distance of 0.5 cm from the branch, and glucose was injected (Figure 3B).

The data acquisition of brain electrical impedance was divided into two parts: (1) After the electrical impedance system was connected to the animal through the electrode line, a blank measurement was performed for 10 min to determine whether the electrode connection was good by observing the size of the one-dimensional impedance value and the sinusoidal waveform of the original data (Xu et al., 2010). (2) After confirming that the electrodes were in good contact and that the connection was stable, carotid perfusion was performed and the start and end frames of angiography perfusion were recorded to observe the changes in brain impedance.

### Data analysis

To further analyze and explore the ability of electrical impedance-enhanced angiographic perfusion to detect intracranial impedance of the normal or occluded unilateral internal carotid artery, we performed a statistical analysis of the reconstructed impedance values from the collected raw voltages. When an animal was given an excitation current with a fixed amplitude, the system collected the original voltage. The data collected by the software were exported, and the forward problem was calculated using an elliptical finite element model that conforms to the actual electrode distribution position, and then the inverse problem was calculated with a damped least squares algorithm and a reconstructed image was obtained., as in Equation 1 (Xu C. et al., 2011).


(1) $$△\,\rho ={\left(J^{T}\,J+\lambda \,R\right)}^{-1}\,J^{T}\,△\,V$$

where △ρ is the change in resistivity distribution at two different time points, *J* is the Jacobian matrix, λ is the regularization parameter (set to five here), *R* is the regularization matrix, and △*V* is the boundary voltage variation vector.

According to anatomical and physiological knowledge, the left and right brains of animals are two relatively independent regions and the unilateral middle cerebral artery is mainly supplied by the ipsilateral internal carotid artery. Therefore, for C-EIT reconstructed images, the left hemisphere is defined as an independent ROI_*left*_ and the right hemisphere as an independent ROI_*right*_. From the contrast agent perfusion monitoring process, we selected a time point every 2 s for a total of eight times (T0 = 0 s, T1 = 2 s, T2 = 4 s, T3 = 6 s, T4 = 8 s, T5 = 10 s, T6 = 12 s, T7 = 14 s) to reconstruct the image. In addition, the average resistivity variation index (ARVI) was calculated for ROI_*left*_ and ROI_*right*_ of the whole process using Equation 2 (Sadleir et al., 2008; Dai et al., 2018), expressed as ARVI_*left*_ and ARVI_*right*_.


(2) $$A\,R\,V\,I=\left(\sum_{i=1}^{N}△\,\rho_{i}\right)/N$$

where △ρ_*i*_ is the reconstructed impedance change for each surface element in the finite element network and *N* is the number of surface elements in the finite element network. A paired-sample *t*-test was used on the impedance change peaks of ROI_*left*_ and ROI_*right*_ in the two groups. In addition, a paired *t*-test was performed to statistically analyze the remodeled impedance values of the left and right cerebral hemispheres in the two groups, and an independent *t*-test was performed to analyze the remodeled impedance values of the left and right cerebral hemispheres between the two groups. Statistical significance was considered *P* < 0.05. The above analyses were from SPSS24 software (IBM Corporation, Armonk, NY, USA).

### Pathological validation

The animals were euthanized in accordance with experimental animal ethics after surgery, and the brains were cut into four coronal slices of 2 mm each. The slices were soaked in 2% 2,3,5-triphenyltetrazolium chloride (TTC) staining solution in a emperature box at 30–37^°^ for 15–30 min. After dyeing, the slices were washed with a phosphate buffer solution and immediately observed and photographed.

In addition, we performed hematoxylin-eosin staining (HE) on the brain tissue of two rabbits in the ICAN and ICAO groups to further microscopically examine the neuronal micromorphology and structure of the left middle cerebral artery. The abdominal aorta was occluded, the left ventricle was impaled, and phosphate buffered saline (150 ml) was continuously perfused, followed by 4% paraformaldehyde (250 ml). The brains were harvested, fixed in 4% paraformaldehyde for 72 h, and wrapped in paraffin. Coronal sections (4 μ m) were prepared, and the morphology of the neurons was observed under a microscope after staining.

## Results

### Pathological results

**FIGURE 4:**
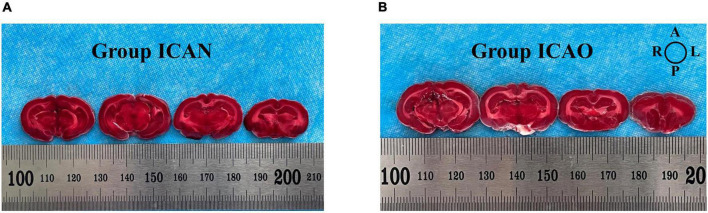
2,3,5-triphenyltetrazolium chloride (TTC)-stained samples from the two groups. **(A)** Indicates the brain tissue in the ICAN group. **(B)** Indicates the brain tissue in the ICAO group. Each slice is 2 mm thick and the slices are arranged from the pineal gland to the olfactory bulb along the sagittal suture. The image orientation is shown in the top-right diagram, where A, L, P, and R represent the anterior, left, posterior, and right parts of the brain, respectively.

**FIGURE 5:**
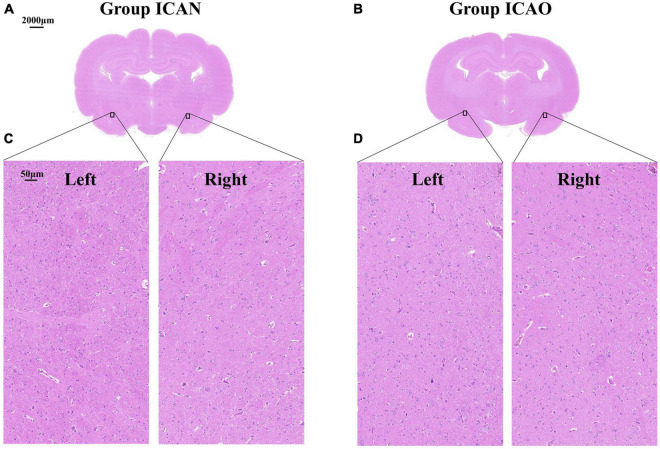
Microstructure of rabbit whole brain tissue under a light microscope at 40 × magnification. **(A,B)** Represent the coronal microstructure of the rabbit brain in the two groups. **(C)** Represents the normal tissue of the left and right cerebral hemispheres of the internal carotid artery non-occlusion (ICAN) group, and **(D)** Represents the brain tissues of the left and right cerebral hemispheres of the internal carotid artery occlusion (ICAO) group, which were the same as the normal brain tissues of the ICAN group.

Figure 4 indicates the TTC results for the ICAN and ICAO groups. Figure 4A indicates the normal brain tissue in the ICAN group (red). Figure 4B shows the brain tissue of the ICAO group, which is also red, indicating that there is no cerebral infarction area. The results of HE staining of the hippocampus of the two groups are shown in Figure 5, with light microscopy at 40 × magnification. After the operation, the rabbit brain tissue in the ICAN group was evenly stained, as shown in Figure 5A, and the ICAO group was also uniformly stained, resembling that of the ICAN group, as shown in Figure 5B. The nuclei and cytoplasm of the left and right cerebral hemispheres were clear in the ICAN group, as shown in Figure 5C. The boundary between the nucleus and cytoplasm of the left cerebral hemisphere in the ICAO group was clear, and most of the cells had intact structures without pyknosis and rupture, as shown in Figure 5D, which was similar to the normal brain tissue in the ICAN group.

### Contrast-enhanced electrical impedance tomography reconstructed image results

**FIGURE 6:**
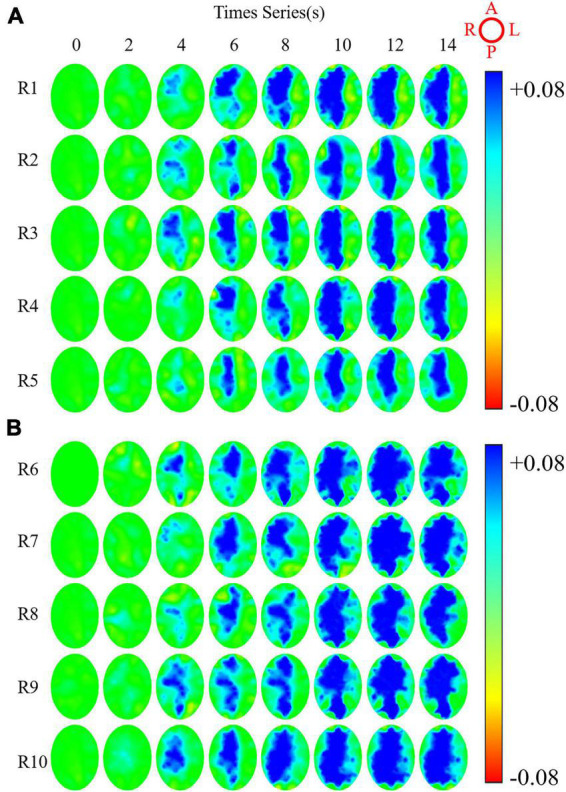
Reconstructed images of rabbits whole brain at eight time points under contrast-enhanced electrical impedance tomography (C-EIT). **(A)** Indicates five rabbits (R1–R5) in the internal carotid artery non-occlusion (ICAN) group. **(B)** Represents five rabbits (R6–R10) in the internal carotid artery occlusion (ICAO) group. The image orientation is shown in the upper right image, A, L, P, R represent the anterior, left, posterior, and right parts of the brain, respectively. The color bar represents the range of possible colors, from increasing electrical resistivity value (blue) to normal baseline intensity (green) and decreasing electrical resistivity value (red).

The C-EIT monitoring data of five rabbits in the ICAN and ICAO groups were randomly selected and the reconstructed images are shown in Figure 6. Figure 6A shows the C-EIT reconstruction images of the ICAN group at each 2 s time point (T1–T8). With the injection of the contrast agent, a blue electrical impedance-enhancing area appeared in ROI_*right*_, and the electrical impedance-enhancing area continued to expand and gradually fill. Figure 6B shows C-EIT reconstruction images of the clamped (ICAO) group at T1–T8. With the injection of the contrast medium, blue areas of electrical impedance enhancement appeared in both ROI_*left*_ and ROI_*right*_. These results indicated that the ICAO group successfully changed the original cerebral blood perfusion pathway. Because of the occlusion of the left common carotid artery, the blood supply to the left side of the rabbit brain was insufficient, and the right side of the rabbit brain provided a compensatory blood supply to the left side of the rabbit brain through the blood supply artery. Among them, the area of electrical impedance enhancement of R8 and R9 at T8 = 14 s was smaller than that at T7 = 12 s, considering that which may be caused by the absorption and dilution of the contrast agent with systemic blood circulation.

### Electrical impedance analysis results

**FIGURE 7:**
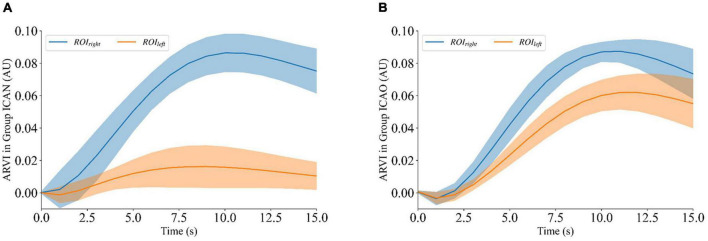
ARVI_*left*_ and ARVI_*right*_ of ROI_*left*_ and ROI_*right*_ during contrast-enhanced electrical impedance tomography (C-EIT) monitoring. **(A)** Indicates that the ARVI_*right*_ of ROI_*right*_ in the internal carotid artery non-occlusion (ICAN) group was significantly increased after the injection of the contrast agent, and the ARVI_*left*_ of the ROI_*left*_ did not change significantly. **(B)** Indicates that the average resistivity variation index (ARVI) of both ROI_*left*_ and ROI_*right*_ in the internal carotid artery occlusion (ICAO) group was significantly increased after injection of the contrast agent.

To quantitatively analyze ARVI_*left*_ and ARVI_*right*_ of ROI_*left*_ and ROI_*right*_ of rabbits after contrast agent perfusion, the experimental data were calculated by intercepting 15 frames from the beginning of the contrast agent injection. Figure 7A shows that the ARVI_*right*_ of the ROI_*right*_ in the ICAN group increased with the perfusion process and gradually decreased to a plateau after reaching the peak at 10 frames, and it remained at the baseline level despite fluctuations of the ROI_*left*_. There was a small increase in ARVI_*left*_ of ROI_*left*_. Considering the small size of the rabbit brain, the measurement data could not be accurately controlled. In the future, animal models with larger brain volumes, such as piglets, should be explored. Figure 7B shows that the ARVI of both ROI_*left*_ and ROI_*right*_ in the ICAO group increased with the perfusion process, and gradually decreased to a plateau after reaching the peak at 10 frames, and the ARVI_*right*_ of ROI_*right*_ was higher than ARVI_*left*_ of ROI_*left*_. This is due to the fact that blood in the left hemisphere is compensated by the right hemisphere through the circle of Willis, so the contrast medium perfusion in the left hemisphere is lower than that in the right hemisphere.

**FIGURE 8:**
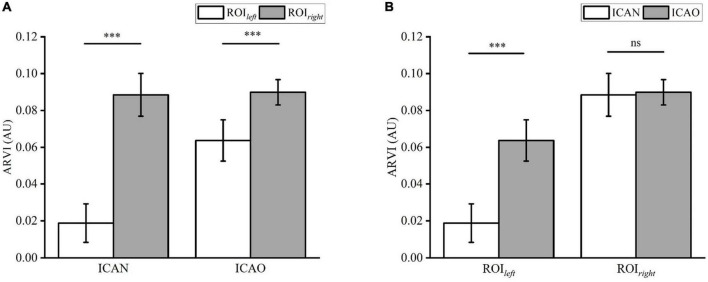
**(A)** Shows the statistical analysis of the reconstructed impedance values of ROI_*left*_ and ROI_*right*_ in the two groups using a paired *t*-test. **(B)** Analysis of the reconstructed impedance values between ROI_*left*_ and ROI_*right*_ of the two groups using an independent *t*-test. (^***^means *p* < 0.001).

Figure 8A shows the results of the electrical impedance changes in the ROI_*left*_ and ROI_*right*_ in the two groups, and the electrical impedance changes in the ICAN group were statistically significant (ICAN-Left: 0.0188 ± 0.0104; ICAN-Right: 0.0885 ± 0.0116; *P* < 0.001). The electrical impedance changes of ROI_*left*_ and ROI_*right*_ in the ICAO group were also statistically significant (ICAO-Left: 0.0637 ± 0.0112; ICAO-Right: 0.0899 ± 0.0069; *P* < 0.001). This is because the left cerebral hemisphere was supplied with blood from the right cerebral hemisphere after the left common carotid artery was occluded in the ICAO group. Owing to the limited blood supply, the electrical impedance of the right cerebral hemisphere was higher than that of the left cerebral hemisphere. Figure 8B shows the results of the electrical impedance changes in the ipsilateral cerebral hemisphere for the two groups. The electrical impedance changes in ROI_*left*_ of the ICAN group and ROI_*left*_ of the ICAO group were statistically significant (ICAN-Left: 0.0188 ± 0.0104; ICAO-Left: 0.0637 ± 0.0112; *P* < 0.001), and there was no statistically significant difference in the electrical impedance changes in the ROI_*right*_ between the ICAN and ICAO groups (ICAN-Right: 0.0885 ± 0.0116; ICAO-Right: 0.0899 ± 0.0069; *P* > 0.05).

The 30 s reconstructed impedance value detected before D5W infusion was used as EIT control (ICAN-Left: 0.0084 ± 0.0025; ICAN-Right: 0.0082 ± 0.0028; ICAO-Left: 0.0096 ± 0.0020; ICAO-Right: 0.0084 ± 0.0038). Compared to the EIT without the contrast agent, injection of 5% glucoses significantly changed the electrical impedance: the *ARVI*_*right*_ of the *ROI*_*right*_ (*P* < 0.001) and the *ARVI*_*left*_ of *ROI*_*left*_ (*P* < 0.05) in the ICAN group; the *ARVI*_*right*_ of the *ROI*_*right*_ (*P* < 0.001) and the *ARVI*_*left*_ of *ROI*_*left*_ (*P* < 0.001) in the ICAO group.

## Discussion

In the study, the feasibility of C-EIT in reflecting cerebral blood perfusion was investigated by monitoring a rabbit model of internal carotid artery embolism. In this experiment, the unilateral internal carotid artery occlusion model was successfully established in ten rabbits, the ICAO group, while the other 10 rabbits with normal internal carotid arteries (ICA) group were monitored by electrical impedance tomography after the model was successfully established. A glucose contrast agent with high blood conductivity was injected into the body through the other internal carotid artery, the collected one-dimensional impedance data were analyzed, and the reconstructed image was reconstructed by the algorithm. The results showed that the impedance value of the left cerebral hemisphere in the ICAN group remained unchanged, while the impedance value of the right hemisphere gradually increased, reached the peak value, and then decreased. According to statistical analysis, there was a statistically significant (*P* < 0.001) difference between the impedance values of the left and right hemispheres. This reflects the cerebral blood flow perfusion in the right cerebral hemisphere. In the ICAO group, the impedance values of both cerebral hemispheres were gradually enhanced, and then began to decrease after reaching the peak value. According to the statistical analysis, there was a significant difference (*P* < 0.001) between the left brain remodeling impedance and that of the right brain. This reflects the compensatory cerebral blood perfusion after diverting some of the original blood supply through the cerebral collateral circulation. Therefore, the C-EIT method proposed in this study is feasible and reflects the state of blood perfusion.

This pilot study was only a qualitative study that explored the feasibility of contrast-enhanced perfusion imaging using cerebral electrical impedance tomography. There has been no comparative study with other existing imaging methods, and it is uncertain whether the cerebral blood perfusion results obtained by C-EIT are accurate. In future research, our research group will not only increase the number of experimental cases but also carry out quantitative research and design other existing means to carry out simultaneous measurements to further verify the effectiveness of this method.

One of the important causes of cerebral ischemia is the occlusion of the ICA, which is a common site of vascular disease (Chen et al., 2021; Hoving et al., 2021). Therefore, a rabbit internal carotid artery occlusion model was selected to replicate clinical internal carotid artery occlusion. When the internal carotid artery, the main blood supply vessel of the cerebral artery, is narrowed or even occluded, collateral circulation of the brain is triggered to ensure an adequate blood supply to the brain tissue, and the blood supply pathway of the cerebral hemisphere on the infarcted side changes (Cuccione et al., 2016). Therefore, internal carotid occlusion can be used as an animal model to analyze cerebral blood perfusion. Sun et al. (2007) established a rat model of subarachnoid hemorrhage to study the effect of *G. biloba* extract on cerebral blood perfusion. Chen et al. (2021) studied the effects of exogenous endothelial progenitor cells on cerebral blood perfusion and microvessels in the injured areas of a rat traumatic brain injury model (Xiao et al., 2013). In the future, our group will explore more cerebral blood perfusion models to better reflect the actual state of cerebral blood perfusion.

Because glucose injection has the advantages of non-conductivity, high contrast with blood conductivity, and a strong reconstructed image signal, glucose injection was selected as the electrical impedance enhancement contrast agent in this study. On the premise of obtaining the patient’s informed consent and not interfering with the normal clinical treatment process, Dai et al. in our research group conducted a clinical dural hematoma drainage test using 5% glucose injection and used EIT to monitor the inflow and discharge process of the glucose solution (Dai et al., 2013). The experimental results indicated that the impedance of the injection area increased significantly with the injection of the glucose solution, indicating that EIT is highly sensitive to intracranial glucose infusion. Therefore, in this study, 5% glucose injection was selected as the contrast agent for brain electrical impedance-enhanced contrast perfusion imaging. In a follow-up study, our team will explore more types of contrast agents, such as normal saline, to achieve better imaging results.

In previous EIT studies, our research group has explored the placement methods of electrodes in various experimental animals and clinical trials. In clinic, the method is non-invasive, in which the patch electrode is directly attached to the scalp (Xu S. et al., 2011; Dai et al., 2013; Li et al., 2018). For large experimental animals, such as piglets, the electrodes were placed on the piglet’s head, and then the hooked copper electrodes were fixed on the piglet’s skull (Dai et al., 2010); For small experimental animals, such as rabbits, electrode placement involves cutting the skin of the cranial dome, followed by exposing the skull and implanting sterile copper electrodes into the skull (Yang et al., 2014, 2017; Dai et al., 2018). Reasons for implantable electrodes in rabbits are as follow: firstly, the skin bleeding may interfere the electrode data collection, resulting in inaccurate measurements of impedance. Secondly, the anatomical size of rabbits’ head is too small to have enough space to fix the sixteen patch electrodes, which could cause unstable measurements and affect the signal quality. Finally, an invasive implantable electrode inside the rabbits’ head can secure the position of the electrode, which can avoid the impact of skin contact on the impedance value. In the future research, we will change the animal model to explore a safer and more reliable electrode placement method for better application.

In this study, arteriography was used as the contrast medium injection method, considering its advantages of stronger enhanced signals and fewer circulatory factors. According to the implementation approach of the existing contrast agent, optional contrast agent injection methods include the internal carotid artery, common carotid artery, cubital vein, and femoral vein. Regardless of whether a vein or artery is selected for contrast medium perfusion, the existing contrast medium can eventually reach the brain through the blood circulation to achieve contrast-enhanced imaging. However, considering that the arteriography perfusion method has the disadvantages of large trauma and inconvenient operation, and we adopted the method of puncturing the internal carotid artery. Hence, this may change the original blood perfusion condition. Therefore, in follow-up research, we will gradually carry out venography exploration experiments with less trauma and a simple operation in combination with different types of contrast agents.

In conclusion, the new cerebral contrast-enhanced electrical impedance tomography method proposed in this study can reflect cerebral blood perfusion through the reconstructed image of the contrast agent, which provides a platform for exploratory research for the detection of cerebral blood perfusion-related diseases. Furthermore, because this method can reflect the distribution of the contrast agent in real time, C-EIT is expected to be used in thrombolytic effect detection, vascular stenosis judgment, collateral circulation evaluation, tumor localization detection, brain function research, and other important applications. Especially in the detection of tumor localization, research on contrast agents that can specifically bind to tumor targets can be further explored. In addition, many other electromagnetic imaging techniques can be adapted to incorporate the principle of differential imaging. In the future, magnetic and magneto-acoustic contrast agents can also be studied, and other electromagnetic and magneto-acoustic imaging technologies can be used for enhanced contrast imaging.

## Data availability statement

The raw data supporting the conclusions of this article will be made available by the authors, without undue reservation.

## Ethics statement

This animal study was reviewed and approved by Experimental Animal Center of Fourth Military Medical University.

## Author contributions

YZ, JY, TZ, WZ, and CX helped design, collect, and analyze data. All authors contributed to this research and subsequent manuscript from conception to final preparation of the article and approved the submitted version.
